# Functional Biomaterials and Biomaterial Composites with Antimicrobial Properties

**DOI:** 10.3390/jfb15090267

**Published:** 2024-09-14

**Authors:** John H. T. Luong

**Affiliations:** School of Chemistry and Analytical & Biological Chemistry Research Facility (ABCRF), University College Cork, College Road, T12 YN60 Cork, Ireland; luongprof@gmail.com

## Antimicrobial Resistance (AMR)

AMR occurs when bacteria, viruses, fungi, and parasites no longer respond to antimicrobial medicines, including antibiotics, antivirals, antifungals, and antiparasitics. AMR increases the risk of disease spread, severe illness, disability, and death. Although it is a natural process that occurs over time through genetic changes in pathogens, AMR is also attributed to the misuse and overuse of antibiotics and has a significant health system and national economic costs. Several drug-resistant bacteria have been identified, such as third-generation cephalosporin-resistant *E. coli*, methicillin-resistant *Staphylococcus aureus*, and *Klebsiella pneumoniae*, a common intestinal bacterium with elevated resistance to critical antibiotics. Multidrug-resistant (MDR) ESKAPE pathogens [1] consisting of *Enterococcus faecium*, *S. aureus*, *K. pneumoniae*, *Acinetobacter baumannii*, *Pseudomonas aeruginosa*, *Enterobacter species*, and extensively drug-resistant (XDR) bacteria have rendered even the most effective and available drugs ineffective.

Significant efforts have focused on natural products with antimicrobial activities, such as flavonoids, tannins, curcumin, lignin, chitosan, natural polymers, and their derivatives, for specific applications [2]. Several nanoparticles (NPs), e.g., silver NPs, zinc NPs, copper NPs, metal–metal oxide composites, etc., also possess strong activity toward a broad range of microbes [3]. Of interest is the antibacterial activity of resveratrol [4] against major foodborne bacteria, including *S. aureus*, *Listeria monocytogenes*, *Camplylobacter jejuni*, and *Vibrio cholera*. Stilbenoids [5] (hydroxylated derivatives of stilbene), polyphenols, and phytoalexins (low-molecular-weight antimicrobial compounds) produced by several plants cause microbial DNA cleavage, membrane damage, decreased cellular metabolic activity, and inhibition of cell division. Other antibiotic alternatives include antimicrobial peptides, cannabinoids, sugar-based bactericides, isoxazole, carbazole, pyrimidine, pyrazole derivatives, essential oils, and anti-quorum sensing agents. In particular, siderophores [6] and prodrugs can serve as excellent platforms for designing new antimicrobial agents against multidrug-resistant bacteria.

These new antimicrobial agents have been advocated for diverse applications ranging from health care, medical textiles, hygiene, medicine, household use, and food packaging. Other medical applications include antimicrobial coatings on implantable devices and medical materials to prevent infection and promote wound healing. As a prerequisite, such antimicrobial compounds must be proven non-toxic to the consumer and cause no allergic, irritating, or sensitizing effects.

The antibacterial activity of novel antimicrobial agents stems from their interactions with bacteria, followed by membrane disruption, enzyme inhibition, and the generation of reactive oxygen species. They can serve as both antibiotics and delivery systems, e.g., nanoparticles. As an example, chitosan-conjugated silver nanoparticles [7] are effective against carbapenem-resistant *A. baumannii* and methicillin-resistant *S. aureus*. Various delivery platforms have been developed, including liposomes, dendrimers, polymeric nanoparticles, and inorganic nanoparticles [8]. They can carry multiple antimicrobial agents or therapeutic payloads to eradicate multiple targets in bacteria simultaneously. Combining antimicrobial agents with conventional antibiotics can result in synergistic effects to enhance the efficacy of antibiotics and decrease the development of bacterial resistance.

To date, different types of nanoparticles have been developed and tested for diverse medical applications. A few examples include mouthwash and nose rinse with AgNP solution, topical silver nanoparticles for microbial activity [9], zinc oxide nanoparticles for the treatment of fungal feet infection [10], ferumoxytol nanoparticles as an antibiofilm treatment [11], a novel respirator with chitosan nanoparticles [12], liposomal amphotericin B [13] for treating patients with invasive fungal infection and SARS-CoV-2 ferritin nanoparticle vaccines with broad SARS coronavirus immunogenicity [14].

However, research efforts are needed to address concerns regarding efficacy, cytotoxicity, and biocompatibility to harness their full potential in controlling antibiotic-resistant bacterial infections and wound management. Studies must be extended from in vitro to in vivo models for the application of antimicrobial agents in medical devices, hygiene products, and other consumer goods. Similar to the use of antibiotics, the mechanisms of antimicrobial properties of any new antimicrobial agent must be fully understood to mitigate this risk while minimizing potential adverse effects. The long-term toxicity and impact on human health and the environment must be fully investigated. New antimicrobial agents may also affect the beneficial microbiota, which play an important role in overall health.

This Special Issue with 10 papers covers a broad subject based on different types of antimicrobial materials. The nanomaterials addressed in this Special Issue include textiles coated with ZnO and TiO_2_ nanoparticles, antifungal activity of biosynthesized silver nanoparticles against *Aspergilli*-causing aspergillosis, antimicrobial and insecticidal selenium nanoparticles, and selenium and tellurium nanoparticles with antibacterial and anticancer potential. Of note are the important biological roles of sulfate radicals coupled with nanomaterials, the antibacterial activity of biomimetic magnesian calcite coatings on biodegradable Mg, and the preparation of whey protein-based films for food packaging. There is growing global research interest in antimicrobial peptides for caries management and antimicrobial biomaterials for the healing of infected bone tissue. In addition to antimicrobial nanomaterials, surface roughness plays an important role in preventing biofilm formation. The design of biomedical surfaces to prevent biofilm formation is another important topic for diverse bioapplications.
